# Exploring college students’ continuance learning intention in data analysis technology courses: the moderating role of self-efficacy

**DOI:** 10.3389/fpsyg.2023.1241693

**Published:** 2023-10-12

**Authors:** Liqiong Liu, Pinghao Ye, Joseph Tan

**Affiliations:** ^1^School of Business Administration, Wuhan Business University, Wuhan, China; ^2^DeGroote School of Business, McMaster University, Hamilton, ON, Canada

**Keywords:** flow experience, self-efficacy, data analysis, continuance learning intention, moderating role

## Abstract

**Introduction:**

In today’s digital economy, data resources have gained strategic recognition. Enterprises view data analytic capabilities as a core organizational competitiveness. This study explored factors influencing college students’ continuance learning intention in data analysis technology courses to inform the role of self-efficacy on the relationship between interactivity and continuance learning intention.

**Methods:**

The research model underpinning the study was based on the Stimulus-Organism-Response model and flow theory. The model was validated using SmartPLS. A total of 314 valid questionnaires were collected via the standard online survey approach.

**Results:**

Among internal factors, study results showed both cognitive interest and self-efficacy had significant positive effects on continuance learning intention. Also, cognitive interest had a significant positive effect on self-efficacy. Among external stimuli, content quality, software quality, and interactivity had significant positive effects on self-efficacy. Software quality did not have a significant effect on cognitive interest. Importantly, self-efficacy registered a significant moderating role on the relationship between interactivity and continuance learning intention.

## 1. Introduction

In today’s digital economy, data resources are strategic assets of enterprises. Informed by multiple studies proving that data analysis (DA) capabilities can significantly improve the performance of manufacturing, retail, and service companies (Feng and Ma, 2022; Jian et al., 2022), Chinese enterprises are viewing DA capabilities as the organization’s core competitiveness.

Lately, Chinese colleges and universities have offered DA technology courses successfully and accumulated preliminary experience in curriculum revamps to meet the need for DA talent among enterprises (Ismail and Haron, 2023). With in-depth training of DA talents and more trending curriculum, attention must now be paid to critical factors motivating students’ learning. Put simply, there is an immediate need to study what impact the curriculum design in revising it to scale up college students’ continuance learning intention in DA technology courses.

Previous studies on continuance intention to learn courses focused primarily on the learning platform of the curriculum. Scholars discussed the continuance intention of learners on different online learning platforms, including mainly the Massive Open Online Course (MOOC) platform (Kim et al., 2021), mobile learning interfaces (Alam et al., 2022) and other digital engagement platforms. Dai et al. (2020), drawing on the Expectation Confirmation Model (ECM), explored the factors underlying the continuance intention to learn on MOOC platform. Their results revealed that although curiosity, the personal trait, was found to largely predict continuance intention, attitude also played a considerably dominant role. Xu and Zhang (2020) found that key factors such as flow experience, perceived mobility and service quality had significant effects on the continuance learning intention of mobile learning platform users. Zhai et al. (2022) built a research model of factors that influenced the continuous use of the AI learning platform of middle school students based on the theory of planned behavior (TPB). Their study showed that subjective norms and perceived behavior control were the direct influencing factors of learners’ continuance intention, while learning utility, learning hedonism, self-efficacy, perceived ease-of-use and accessibility indirectly affected learners’ continuance intention.

Some other studies had directly focused on the intention to continue learning in specific courses. Wang et al. (2022) studied the continuous learning intention of online mental health courses of college students in the context of the COVID-19 epidemic, and concluded that perceived value had a positive impact on the continuance learning intention. Tan and Fu (2020) constructed a modified model on the factors affecting online English continuance learning intention of college students. Their research explored the multi-relations among the perceived usefulness, confirmation, satisfaction and continuance learning intention of online English learning. Their results showed that college students had a higher online English learning satisfaction and continuance learning intention. The perceived usefulness and expectation confirmation could directly affect the continuous online English learning intention of college students.

In conclusion, we can make some observations. First, in terms of research objects, it was common to discuss the continuance learning intention of language and mental health courses. Even so, despite its growing importance and urgency, there is scanty research on DA technology courses. Second, in terms of research content, the influencing factors of college students’ intention to continue learning courses mostly included learner factors and learning environment factors. Learner factors such as learners’ individual experience (Lin, 2011; Diotaiuti et al., 2021), self-efficacy (Zhai et al., 2022), hedonism (Wang et al., 2019) and perceived entertainment (Roca and Gagné, 2008) were thought to have an effect on their continuance learning intention. Moreover, past relevant research also concentrated on the pressure brought by external environment on these learners, in particular, those manifested in social influence, subjective norms, social cognition, and beyond (Chen, 2022; Yang et al., 2022; Jiang and Liang, 2023). In addition, teacher support and form of learning would also affect the continuance learning intention (Wu et al., 2020). Third, in terms of research methods, scholars mainly used ECM (Chow and Shi, 2014; Rekha et al., 2022) and TPB (Zhai et al., 2022) to analyze the relationship between expectation, perceived performance, perceived usefulness, perceived ease-of-use and the continuance learning intention.

Moreover, developing talents in DA ability requires that college students do have the motivation and willingness to further study and practice, following learning DA technology courses. This leads to the need to clarify key influencing factors of college students’ continuance learning intention. DA technology courses, which support DA capabilities (Huang and Li, 2016), can cultivate college students’ ability to solve economic management problems. Notwithstanding, students often find such courses difficult to absorb. Beyond external stimuli, learners’ motivation is also closely linked to an individual’s flow experience. Accordingly, the S-O-R (Stimulus-Organism-Response) model is employed in the current study to explore the relationship between internal-external factors and college students’ continuance learning intention in DA technology courses. Not only do this study contributes to extend the research boundary of flow experience, but it is intended to also enrich the extant literature on continuance learning intention, providing a theoretical basis for colleges and universities to offer students educational psychological counseling of curriculum learning, and laying a foundation for scaling up the talent pool with DA capabilities for the greater good of society.

## 2. Theoretical basis

### 2.1. The S-O-R model

To explain the psychological decision-making process of individuals after being stimulated by the external environment, Mehrabian-Russel proposed the S-O-R model to point out that stimulation (Stimulus) from the external environment will affect an individual’s cognitive status (Organism), thereby changing their behavioral response (Response).

The S-O-R model is widely used to understand consumer behavior (Han et al., 2022). For example, it has proven to explain individual behavior motivations effectively (Zhou et al., 2022). In recent years, educators have also used it to predict learners’ learning behavior and intention (Zhao et al., 2020). Specifically, we use the S-O-R model here to analyze the effect path and mechanism of college students’ continuance learning intention in DA technology courses. In this context, “stimulus” comes from the external environment, acting as an external cause. College students’ learning behavior in DA technology courses are affected at the same time by factors such as courses, software, peers, and other variables. More specifically, this research incorporates content quality, software quality, and interactivity into the index system as the three key aspects of the stimulus. “Organism” refers to an individual’s internal cognition and attitude after receiving the external stimuli and may be regarded as an internal cause. Here, organism is represented by the cognitive interest and self-efficacy. “Response” is an individual’s decision formed following the action of internal-external factors, which refers here to the continuance of learning intention.

### 2.2. Flow experience

Csikszentmihalyi (1975) first proposed the concept of flow experience from the perspective of psychology, believing that flow experience is the process of optimal experience and the overall feeling of an individual fully involved in a certain activity.

Over the years, flow theory has gradually evolved. Early scholars have classified flow experience into three categories: (1) the preconditions for the generation of flow experience; (2) the cognitive characteristics of the flow experience or process; and (3) the results of flow experience (Hoffman and Novak, 1996). When learners have flow experience, the kinetic energy of learning increases, and they are more inclined to learn continually. In this research, the two elements of cognitive interest and self-efficacy in flow experience have therefore been introduced as factors that affect college students’ intention to continue learning DA technology courses.

### 2.3. Continuance learning intention

Research on continuance intention first appeared in information systems (IS), where scholars systematically defined and discussed continuance usage intention (Bhattacherjee, 2001b). The user’s experience with using a certain IS for the first time will affect their intention to reuse or continually use a system. With respect to the field of education, learners’ prior learning experiences will affect their subsequent learning intentions and behaviors. Continuance learning intention refers to a learner’s intention to complete the current learning task or to continue participating in the next course (Joo et al., 2010). This study contextually refers to a learner’s continuance intention to carry out a certain learning behavior in the future and the intention to recommend it to others.

To date, scholars have accumulated some research results in terms of continuance learning intention, but the research objects mainly involve language courses, mental health courses, and other business courses. Sadly, research on DA technology courses is scanty. In reviewing prior research driven by the S-O-R model, few researchers have studied cognitive interest and self-efficacy as internal factors from the perspective of flow experience. While there is a small body of literature exploring the moderating role of self-efficacy, there still is plenty of room for future research on the continuance learning intention. Accordingly, this study explores the key factors and mechanisms that affected college students’ continuance learning intention in DA technology courses and further validates the moderating role of self-efficacy on the relationship between interactivity and continuance learning intention.

## 3. Research model and hypotheses

**Figure 1:**
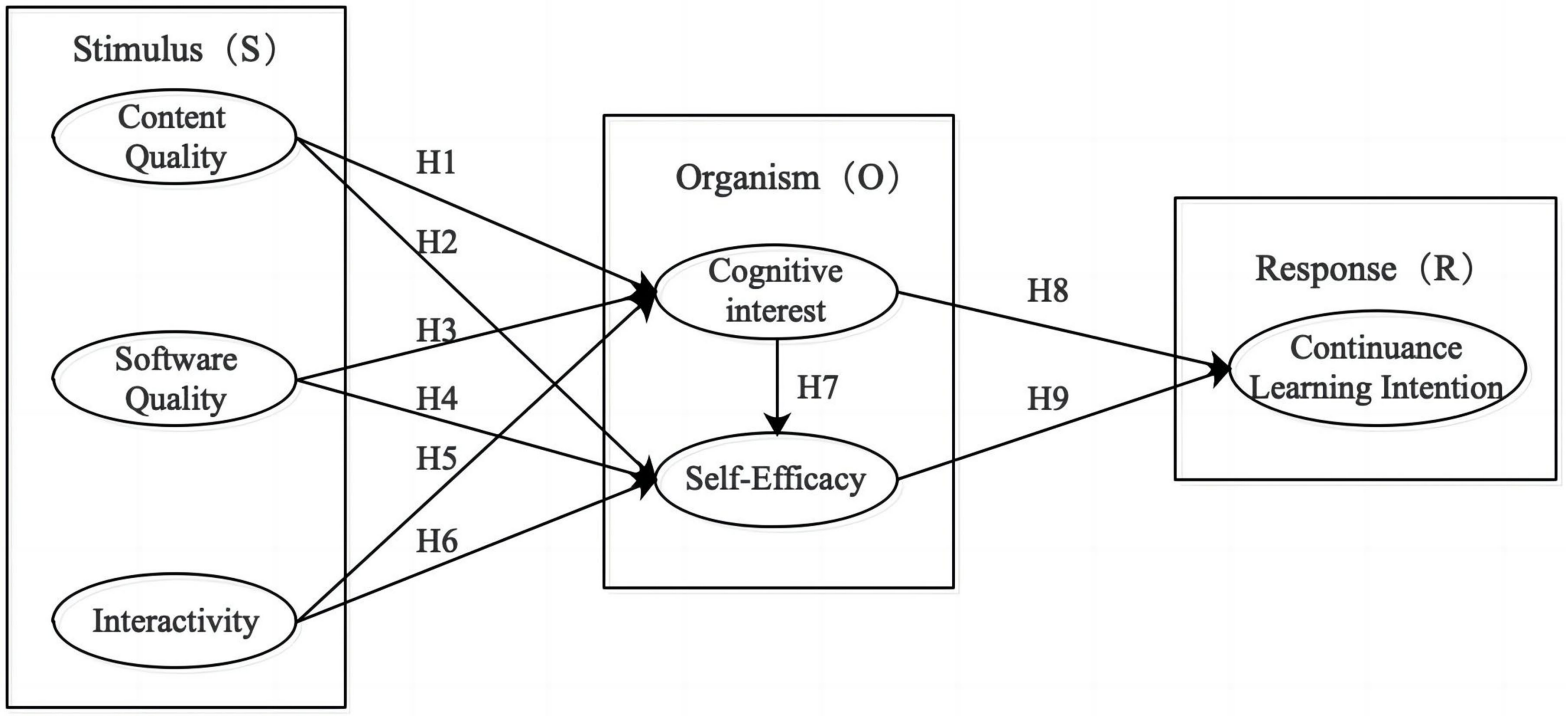
Research model.

Figure 1 depicts the underlying S-O-R model and flow theory directing efforts in the current study.

### 3.1. (Stimulus – S): content quality, software quality, and interactivity

A complete course structure characterizes the content quality of DA technology courses, highlights key points and difficult concepts, and aggregates a proper amount of practice exercises to facilitate learners to consolidate what they have learned. The content quality will affect the learning experience of college students because high content quality contributes to a better learning experience and continuance learning intention.

Prior studies had proved that the content quality of courses could affect the satisfaction or user perception of learners. Purportedly, such satisfaction or perceived value of learners would also positively affect the learners’ continuance learning intention (Roca et al., 2006; Wang et al., 2022). The optimal state of learning can produce a flow experience. The cognitive characteristics of college students in immersive learning include cognitive interest and self-efficacy. In this study, we define learners’ interest and psychological tendency in learning courses as cognitive interest; similarly, we define the learners’ confidence in their ability in learning courses as self-efficacy. The following study hypotheses are therefore advanced:

*H1:* Content quality has a significantly positive effect on cognitive interest.

*H2:* Content quality has a significantly positive effect on self-efficacy.

Learners will use platforms, systems, technologies, or software during learning. DA technology courses usually use SPSS/SAS/SAP or R/Python software.

Software quality refers to software stability, timely version updates, and the ability of software to aid users effectively. Higher software quality leads to a better learning experience, promoting continuous learning intention. Researchers had found that system quality can positively affect learners’ continuance e-learning intention vis-à-vis the users’ satisfaction (Ismail et al., 2012). Therefore, the following study hypotheses are also inferred:

*H3:* Software quality has a significantly positive effect on cognitive interest.

*H4:* Software quality has a significantly positive effect on self-efficacy.

Interactivity refers to the amount of communication between college students and their teachers and/or classmates during the learning process. In the context of DA technology courses learning, learners are often prone to doubt and tend to slack. Feedback from others (teachers and/or classmates) can help resolve problems, enhancing the continuance learning intention. Empirical studies had shown interactivity, a common study variable of continuance learning intention, to positively affect learners’ continuance learning intention through experiential mediator variables such as perceived usefulness, fun, immersion, and more (Zhang, 2016; Zhu, 2017). Therefore, the following study hypotheses are projected:

*H5:* Interactivity has a significantly positive effect on cognitive interest.

*H6:* Interactivity has a significantly positive effect on self-efficacy.

### 3.2. (Organism – O): cognitive interest and self-efficacy

When college students are learning a DA technology course, a clear understanding of the course and an interest in it can help them properly assess their abilities to perform specific tasks, thereby building confidence in learning. Using questionnaires, several researchers had found that children’s interest in specific types of writing is closely related to self-efficacy (Hidi et al., 2002). Others had also reported that parental participation, academic interest, and school environment could significantly and positively affect junior high school students’ academic self-efficacy (Adeyemo, 2005). Therefore, the following study hypothesis is advanced:

*H7:* Cognitive interest has a significantly positive effect on self-efficacy.

### 3.3. (Response – R): continuance learning intention

This study defines continuance learning intention as the learner’s continuance intention to carry out a certain learning behavior in the future and the intention to recommend it to others.

Cognitive interest and self-efficacy are internal factors that affect the individual’s continuance learning intention. Generally, the higher the cognitive interest and self-efficacy of learners, the stronger their continuance learning intention. Previous research findings had proved that learning interest positively correlates with the continuance learning intention in MOOCs. At the same time, it was claimed that enhancing learners’ metacognition could help improve online learning interest and continuance of learning intention (Tsai et al., 2018). Shao (2018) discovered that learners’ self-efficacy at the individual level would affect MOOC learners’ continuance learning intention through perceived ease-of-use. Therefore, the following study hypotheses are proposed:

*H8:* Cognitive interest has a significantly positive effect on continuance learning intention.

*H9:* Self-efficacy has a significantly positive effect on continuance learning intention.

### 3.4. Moderating role of self-efficacy

Researchers had also explored the influence or moderating role of self-efficacy on continuance learning intention in previous studies. Moreno et al. (2017) for example, had shown system interactivity, cognitive absorption, self-efficacy, and facilitating conditions have significantly affected students’ intention to use e-learning platforms. The Mohamad-Rahim study unveiled that online self-efficacy moderates enjoying MOOCs and continuance learning intention (Mohamad and Rahim, 2018). On continuance intention, Maawaddah and Yin (2020) had proposed online self-efficacy as a moderating variable to better understand the influence of individual differences and MOOC characteristics (usefulness, enjoyment, interactivity, and openness). Accordingly, self-efficacy can be seen as tending to act as a moderator between interactivity and continuance of learning intention. Therefore, the following study hypothesis is contemplated:

*H10:* Self-efficacy has a significant moderating role on the relationship between interactivity and continuance learning intention.

## 4. Research methods

### 4.1. Sample and data collection

The present research studies college student learners and uses questionnaires to gather data toward testing and validating the series of study hypotheses noted in Section 3. The questionnaires had been distributed online through WJX, a well-known online questionnaire survey platform in China, following unified standards and requirements.

Three hundred fifty-five (355) questionnaires had been distributed, yielding a total of three hundred fourteen (314) valid questionnaires being responded, with a response rate of 88.5%. Descriptive resulting statistics showed that among the respondents, males accounted for 39.2% and females 60.8%. With respect to educational grade levels, freshmen accounted for 12.1%, sophomores 26.4%, juniors 42.7%, and seniors 18.8%. In terms of type of study major, 52.9% were in liberal arts, 14.6% in science, 22.0% in engineering, and 10.5% in other majors. Finally, 32.5% of the respondents took one DA technology course, 43.3% took two courses, 18.5% took 3 courses, and 5.7% took 4 or more DA technology courses. The general information on the respondents is shown in Table 1.

**Table 1:** Demographic characteristics of valid samples.

Variables	Categories	Frequency	Percentage
Gender	Male	123	39.2%
	Female	191	60.8%
Grade	Freshman	38	12.1%
	Sophomore	83	26.4%
	Junior	134	42.7%
	Senior	59	18.8%
Type of Major	Liberal arts	166	52.9%
	Science	46	14.6%
	Engineering	69	22.0%
	Other	33	10.5%
Number of courses enrolled	1	102	32.5%
	2	136	43.3%
	3	58	18.5%
	4 or more	18	5.7%

### 4.2. Questionnaire and measurements

The measurement questionnaire in this study had been formulated based on mature scales, and the research design strictly followed the “translation-back translation” procedure. Appropriate adjustments had also been made in the Chinese context. At the same time, college students were interviewed on the comprehensibility of the problem description. A pre-survey questionnaire was distributed, and the questionnaire was further modified and improved to its final version based on the feedback from the pre-survey. The questionnaire used a Likert five-point scale ranging from 1 = strongly disagree to 5 = strongly agree.

In this study, the questionnaire had been carefully revised with the characteristics of the DA technology course considered according to the scale of external stimuli such as interactivity compiled by Filep and Qian Ying. Based on the feedback and pre-survey results, the scales of the three latent variables of content quality, software quality, and interactivity were finally determined. This study drew on the scale compiled by Guo Xiuwei and Li et al. to form a scale of internal factors (cognitive interest and self-efficacy). This research also drew on the scale compiled by Bhattacherjee et al. to measure the continuance learning intention. The measured items and sources are shown in Table 2.

**Table 2:** Model measurement questionnaire.

Construct	Coding	Measurement item	Sources
Content Quality (CQ)	CQ1	The course is well structured, and the chapters are arranged in proper order, making it easier to learn.	Lin and Wang (2012), Mclean (1992)
	CQ2	The key points and difficult points of the course are highlighted, making it easier to learn.	
	CQ3	The course provided just the right amount of exercises to help me retain learning.	
Software Quality (SQ)	SQ1	The software used in the data analysis technology course is stable.	Wu and Wang (2006)
	SQ2	The software used in the data analysis technology course is updated promptly.	
	SQ3	The software used in the data analysis technology course provides an effective Help function.	
Interactivity (IN)	IN1	During the learning process, I have the opportunity to interact with the teacher.	Filep (2009), Qian (2015)
	IN2	During the learning process, the teacher will answer my questions.	
	IN3	During the learning process, my classmates can always give me timely feedback.	
Cognitive Interest (CI)	CI1	I am interested in the content of the data analysis technology course.	Guo (2007)
	CI2	I experienced the joy of learning while studying.	
Self-Efficacy (SE)	SE1	My ability meets the requirements of the data analysis technology course.	Li et al. (2015)
	SE2	I am confident that I can understand the content of the course that I have studied.	
	SE3	I am confident that I can complete the learning tasks assigned by the teachers of the data analysis technology course.	
	SE4	I can solve problems encountered in the course-learning process.	
Continuance Learning Intention (CLI)	CLI1	I will study other data analysis technology courses in the future.	Bhattacherjee (2001a), Lin and Wang (2012)
	CLI2	I will use data analysis knowledge to solve problems in my study and work in the future	
	CLI3	I would like to recommend the data analysis technology course to my friends.	

### 4.3. Reliability and validity

SPSS24.0 was used to test the reliability-validity of content quality, software quality, interactivity, cognitive interest, self-efficacy, and continuance learning intention. Results where tabulated and summarized below.

Table 3 shows that the composite reliability (CR) of each latent variable to be greater than 0.78, and the Cronbach’α coefficient of each variable to be greater than the minimum acceptable level of 0.6, indicating that the scales have good reliability. The exploratory factor analysis method had been used to test the structural validity of the scale. The factor loading of the item corresponding to each variable was found to be greater than the threshold value of 0.7, indicating good structural validity of the scale (Nunnally, 1978).

**Table 3:** Reliability analysis.

Construct	Coding	Factor loading^a^	Cronbach’s a	CR	AVE
Content quality (CQ)	CQ1	0.775	0.719	0.814	0.594
	CQ2	0.756			
	CQ3	0.781			
Software quality (SQ)	SQ1	0.738	0.651	0.786	0.550
	SQ2	0.771			
	SQ3	0.715			
Interactivity (IN)	IN1	0.745	0.643	0.78	0.542
	IN2	0.759			
	IN3	0.704			
Cognitive interest (CI)	CI1	0.850	0.705	0.849	0.738
	CI2	0.868			
Self-efficacy (SE)	SE1	0.769	0.757	0.845	0.578
	SE2	0.732			
	SE3	0.793			
	SE4	0.746			
Continuance learning intention (CLI)	CLI1	0.767	0.680	0.785	0.550
	CLI2	0.723			
	CLI3	0.733			

The average variance extracted (AVE) of each variable was found to be greater than 0.5, indicating good convergent validity of the scale (Fornell and Larcker, 1981). Table 4 shows that the square root of AVE (SRAVE) of each variable to be greater than the correlation coefficient between this and other variables, and the variables had good discriminant validity, proving that the scale used in this study has good validity.

**Table 4:** Validity analysis.

	CQ	SQ	IN	CI	SE	CLI
CQ	**0.771**					
SQ	0.486	**0.742**				
IN	0.485	0.473	**0.736**			
CI	0.455	0.371	0.486	**0.859**		
SE	0.524	0.456	0.489	0.608	**0.760**	
CLI	0.495	0.360	0.491	0.601	0.546	**0.741**

Values on the diagonal are the square root of the AVE.

### 4.4. Common method bias and non-response bias

Given that the collected data had all been derived from questionnaires filled out by respondents, the validity of the data might be affected by common method biases. In order to eliminate this hidden danger, homologous variance test has been performed on the data using Harman’s single factor test method (Podsakoff and Organ, 1986).

Test results showed that the first factor explained 33.79% of the total variance in the data without rotation, which did not exceed 50% of the recommended value (Podsakoff et al., 2003), indicating that common method bias did not affect the validity of the conclusions.

To exclude the possible influence of non-response bias, the respondents had also been subdivided into two groups based on the time they had participated in the survey. Following the method of previous scholars (Ma and Agarwal, 2007), no differences were found between the two subsamples by comparing the demographics of early v. late participants. Furthermore, the Chi-square test unveiled that there was no statistical difference between the two groups in the weight of model measurement. Put simply, the data have no non-response bias.

## 5. Results

This study uses the partial least squares (PLS) method to analyze the model. The PLS method is a new multivariate data analysis method. Compared with other methods, it can obtain more reliable and stable results, making it suitable for small samples.

Additionally, PLS can simultaneously accomplish predictive modeling, comprehensive simplification of the multivariate system, and correlation analysis between two groups of variables. It can effectively solve the problem of collinearity. Its main purpose is to construct the regression model between multi-dependent and multi-independent variables (Chin et al., 2020). When the model is being constructed, the PLS method can flexibly set the type of external relations in the structural equation according to the actual situation; that is, it supports the constitutive and reflective models (Richter et al., 2020). Specifically, the study used Smart PLS3.0 software to analyze the model.

### 5.1. Path coefficient and hypothesis test

**Figure 2:**
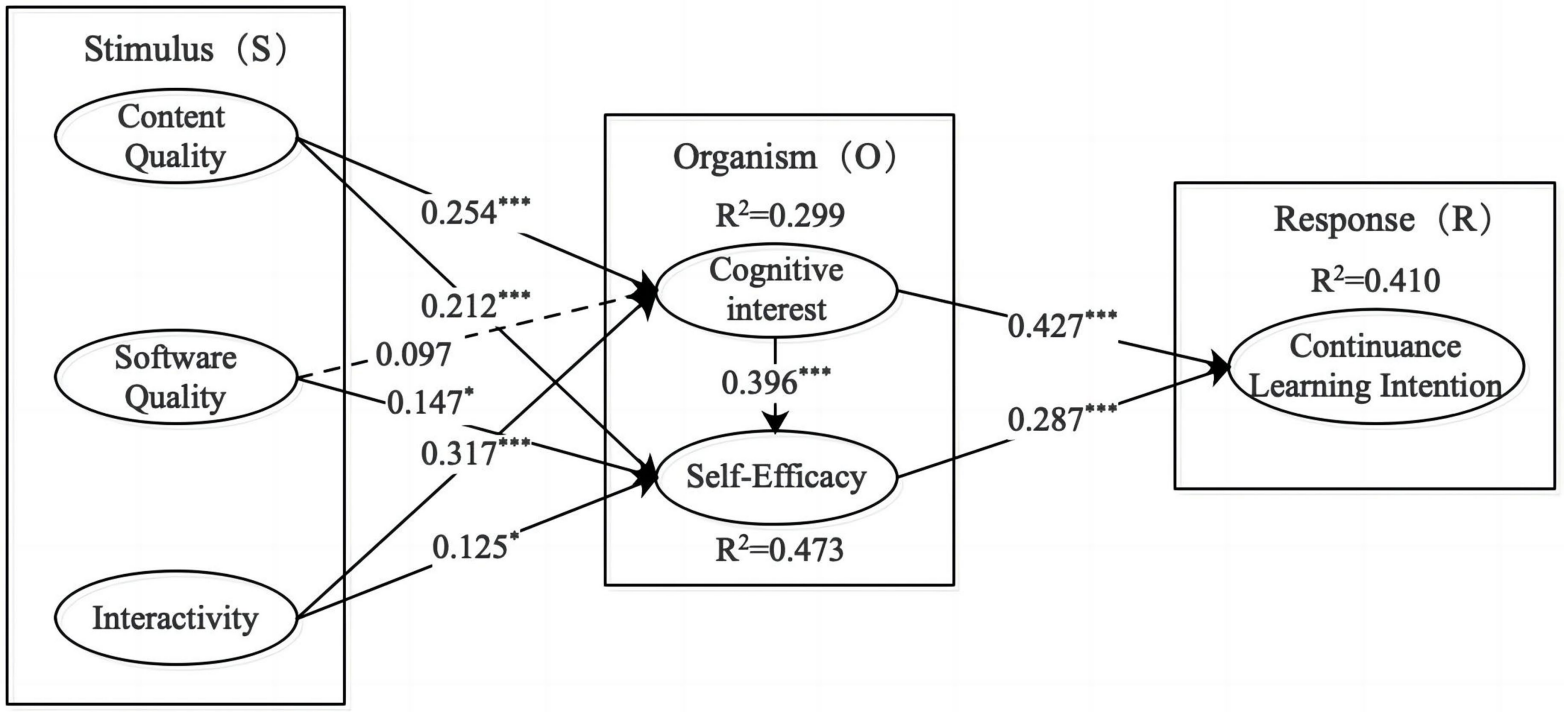
Model path and significance level. ***p < 0.001, *p < 0.05; dotted line indicates that the hypothesis is rejected.

The path coefficient indicates the strength of the relationship between the independent and dependent variables (Thom, 1983). The path coefficient analysis results of the research model are shown in Table 5 and Figure 2, and across all tested hypotheses, only *H3* hypothesis failed to hold.

**Table 5:** Hypothesis testing result.

Hypothesis	Path	Means	SD	*t*	*p*	Result
*H1*	CQ → CI	0.254	0.065	3.933	0.000	Supported
*H2*	CQ → SE	0.212	0.064	3.330	0.001	Supported
*H3*	SQ → CI	0.097	0.066	1.475	0.152	Not supported
*H4*	SQ → SE	0.147	0.058	2.532	0.014	Supported
*H5*	IN→CI	0.317	0.058	5.475	0.000	Supported
*H6*	IN→SE	0.125	0.058	2.134	0.034	Supported
*H7*	CI → SE	0.396	0.058	6.803	0.000	Supported
*H8*	CI → CLI	0.427	0.050	8.552	0.000	Supported
*H9*	SE → CLI	0.287	0.055	5.196	0.000	Supported

*R*^2^ represents the proportion of variance in the dependent variable explained by the independent variables. This study used the bootstrapping repeated sampling method to select 3,000 samples to calculate the *t* value of the significance test. The explanatory degrees of cognitive interest, self-efficacy, and continuance learning intention, respectively, were 0.299, 0.473, and 0.410, indicating the model has good explanatory power.

The bootstrapping repeated sampling method had been used to test the significance of the path coefficient of the structural model. Table 5 shows that content quality had a significantly positive effect on cognitive interest (*β* = 0.254, *t* = 3.933), confirming *H1*. Its positive effect on self-efficacy was also significant (*β* = 0.212, *t* = 3.330), thus confirming *H2*. The effect of software quality on cognitive interest had not been proven (*β* = 0.097, *t* = 1.475), and thus, *H3* is rejected. Software quality affected self-efficacy (*β* = 0.147, *t* = 2.532) significantly and positively, thereby validating *H4*.

Interactivity significantly positively effected cognitive interest (*β* = 0.317, *t* = 5.475) and self-efficacy (*β* = 0.125, *t* = 2.134), confirming *H5* and *H6*. Cognitive interest affected self-efficacy (*β* = 0.396, *t* = 6.803) and continuance learning intention (*β* = 0.427, *t* = 8.552) in a significantly positive way, so *H7* and *H8* were also supported. Self-efficacy had a significantly positive effect on continuance learning intention (*β* = 0.287, *t* = 5.196), and thus, *H9* is confirmed.

### 5.2. Moderating role test

This study used the hierarchical regression analysis to test the moderating role of self-efficacy on the relationship between interactivity and continuance learning intention. Before testing the moderating role, it was necessary to center the variables of the cross term to avoid collinearity and then multiply the centered variables to construct the interaction term.

In this research, the independent and moderating variables had been centered to obtain the product terms of interactivity and self-efficacy. Table 6 shows results of the subsequent multilevel regression analysis and indicate that self-efficacy significantly moderated the relationship between interactivity and continuance learning intention (*β* = −0.147, *t* = −1.995, *p* = 0.047 < 0.05).

**Table 6:** Moderating role of self-efficacy on the relationship between interactivity and continuance learning intention.

	Coefficient	SE	*t*	*p*	LLCI	ULCI
Constant	4.102	0.028	145.552	0.000	4.047	4.157
IN	0.297	0.053	5.557	0.000	0.192	0.402
SE	0.368	0.049	7.590	0.000	0.273	0.464
IN*SE	−0.147	0.074	−1.995	0.047	−0.291	−0.002

The study divided self-efficacy into high v. low to showcase its role as a moderating variable. MS Excel had been used to draw the effect of interactivity on continuance learning intention under the high v. low self-efficacy scenarios.

The main effect (interactivity) was found to be 0.297, the moderating variable (self-efficacy) effect 0.368, and the moderating effect (interactivity × self-efficacy) −0.147, *p* = 0.047.

**Figure 3:**
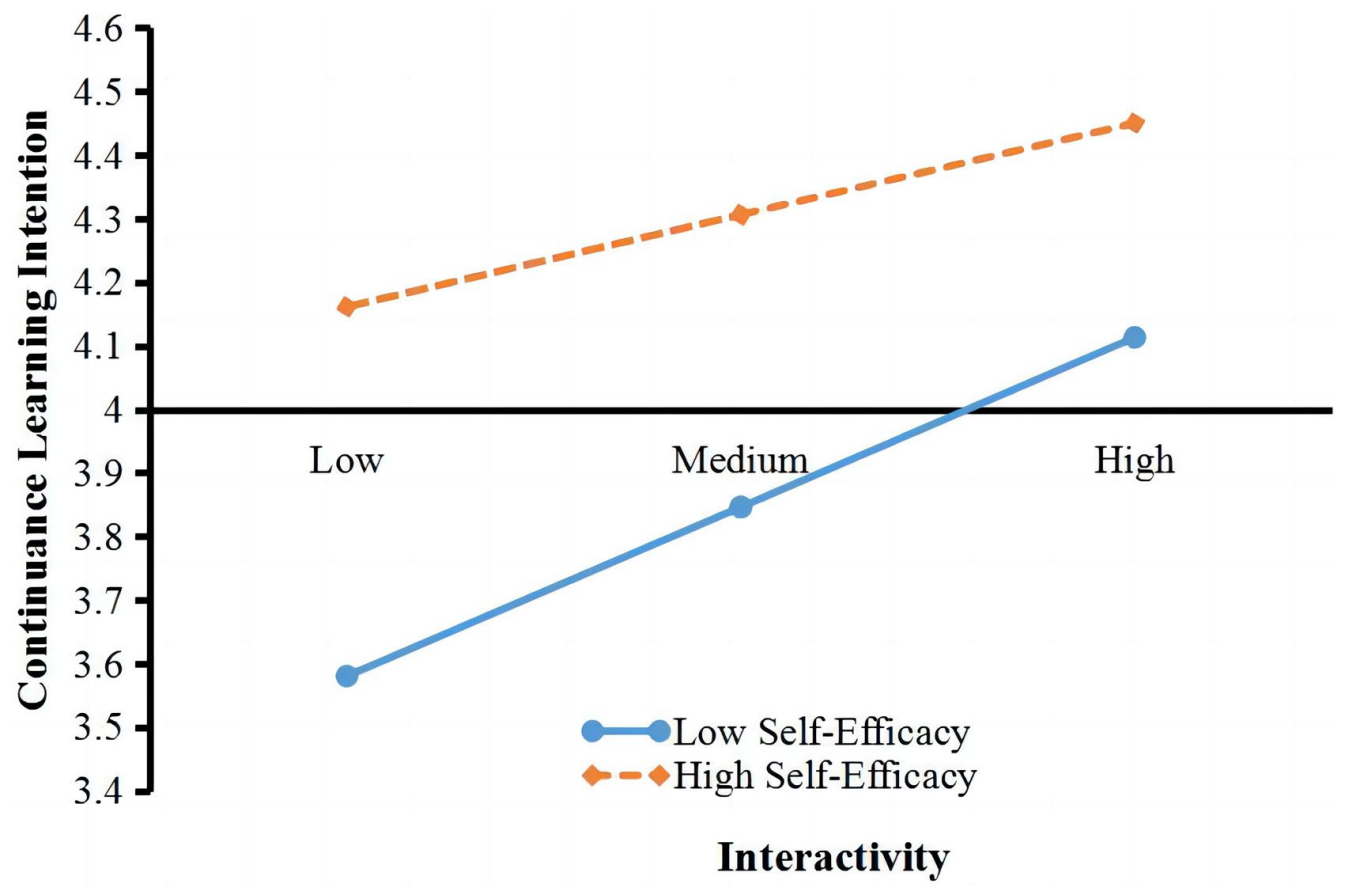
Moderating role of self-efficacy on the relationship between interactivity and continuance learning intention.

Results of the moderating role, as shown in Figure 3, indicated that under high self-efficacy, the effect of interactivity on continuance learning intention was weakened, and hence, *H10* is confirmed.

## 6. Discussion

### 6.1. Discussion of the findings

#### 6.1.1. Analysis of the relationship between external stimuli and internal factors

Regarding S, the external stimuli, both content quality and interactivity positively affected cognitive interest. These findings are consistent with previous ones (Chen, 2017; Wang et al., 2022). A well-structured DA technology course that provides summary of key points, highlights difficult concepts, and offers an appropriate number of thought-provoking exercises makes it easier for college students to generate cognitive interest while learning. Learners would feel useful and joyful (Lin and Wang, 2012). Notably, previous empirical contributions had also shown interactivity to positively affect learners’ continuance learning intention through experiential mediator variables such as perceived usefulness, fun, immersion, and more (Zhang, 2016; Zhu, 2017). While learning, having the ability to engage and interact with teachers and/or other classmates in a timely manner will further enhance college students’ interests and learning behaviors in DA technology courses.

Nonetheless, the positive effect of software quality on cognitive interest could not be validated, which surprised us as it is inconsistent with the hypothesis. Indeed, some recent studies on online learning have demonstrated system quality to encourage cognitive interest (Wen et al., 2023). A plausible explanation here is that discrepancies among different platforms used in online learning, as an emerging learning modality, may have led to mixed feeling with the use of different functions associated with its learning platform; therefore, system quality, such as stability, convenience, and fluency, can all have differential impact on learners’ cognitive interest. Even so, software used by colleges in the current study have been relatively mature with stable functions. Hence, it may be the lack of timely version updates of software, and other factors that impact on the intensity of college students’ cognitive interest, which need further investigation. Hence, future studies must begin to pay more attention to the change of education mode (Mittal et al., 2021; Sethi et al., 2022; Tandon et al., 2022) and the quality of online learning platforms (Tseng, 2020).

Moreover, with reference to external stimuli (S), it was found that content quality, software quality and interactivity could have significant positive effects on self-efficacy. These S factors could effectively improve college students’ belief in their ability to learn DA courses. This is consistent with previously reported findings and the current study added to the research by showing that content quality, software quality and interactivity are directly related to self-efficacy.

#### 6.1.2. Analysis of the relationship between internal factors and self-efficacy

In reference to internal factors (O), our study results showed that cognitive interest and self-efficacy had a significant positive effect on continuance learning intention, and cognitive interest also had a significant positive effect on self-efficacy. Indeed, cognitive interest and self-efficacy significantly affected continuance learning intention positively, which in combination explains 41.0% of the variance of continuance learning intention. The effect of cognitive interest on continuance learning intention was extremely prominent (Tsai et al., 2018), indicating that interest is still the best teacher when college students are learning DA technology courses.

Learners’ interest in the content and form of learning and their behavioral tendency in learning can help to improve the continuance of learning intention and, at the same time, enhance the individual’s sense of self-efficacy. Self-efficacy is also an essential factor in college students’ continuance learning intention (Hu, 2014; Shao, 2018). Briefly, the stronger the self-efficacy, the stronger the learners’ confidence in their learning ability, and the more likely they will persist in learning DA technology courses. As indicated by previous scholars (Adeyemo, 2005), cognitive interest had a significant positive impact on self-efficacy. While exploring the continuance learning intention of distance education learners, some researchers have found that cognitive interest positively affected self-efficacy, which also affirm the aforenoted finding (Ying, 2021).

#### 6.1.3. Analysis of the moderating role of self-efficacy on the relationship between interactivity and continuance learning intention

As for the role of self-efficacy, this study unveiled a significant moderating role of self-efficacy on the relationship between interactivity and continuance learning intention. This revelation is consistent with recent findings (Maawaddah and Yin, 2020).

Hence, the current study harmonizes with previous ones by clarifying the moderating role of self-efficacy on the relationship between interactivity and continuance learning intention. Under high self-efficacy, the effect of interactivity on continuance learning intention was weakened. A reasonable explanation is that when college students are confident in their learning ability, their intrinsic motivation will weaken their dependence on external interactions. Therefore, under high self-efficacy, the effect of interactivity on continuance learning intention was weakened. Altogether, compared with S factors, the O factors appeared to have more obvious effects on continuance learning intention.

This study did not find that self-efficacy had a significant moderating effect on the relationship between content quality or software quality and continuance learning intention. After reviewing the existing literature, an attempt was made to uncover the reasons. Wang et al. (2022), for example, revealed that the quality of knowledge content was one of the main factors when discussing the factors affecting college students’ intention to continue learning in mental health courses on online MOOC platforms. In addition, according to Zhao et al. (2018), perceived control, perceived response, and perceived mutual aid indirectly affected the continuance learning intention through social presence. Perceived control refers to the learner’s perception of technical attributes, similar to software quality in this study; in contrast, perceived response and perceived mutual aid are comparable to the interactivity construct of this study. It can be reasonably inferred then that content quality had a greater effect on college students’ continuance learning intention than interactivity, so self-efficacy could not significantly regulate the relationship between content quality and continuance learning intention. The role of software quality could be more complex and might involve other reasons. It should be further explored in future studies.

### 6.2. Theoretical contributions

By hypothesizing and verifying the relationship between (S) external stimuli, (O) internal factors, and (R) continuance learning intention, several theoretical contributions may now be drawn from the findings of the current study.

First, this study complements the limited literature about college students’ continuance learning intention in the context of DA technology courses. Based on ECM (Dai et al., 2020; Zhao et al., 2022) and TAM (Wu and Chen, 2017), scholars have proved that individual factors and environmental factors can promote learners’ continuance learning intention. However, the research objects mainly involve language courses, mental health courses, and business courses (Alam et al., 2022; Cai and Wang, 2022; Wang et al., 2022), whereas the research on DA technology courses is lacking. Based on the S-O-R model, this study filled the missing gaps with respect to DA technology courses on key influencing factors affecting college students’ continuance learning intention; more specifically, the findings not only aided to clarify the influence path and mechanism of internal v. external factors, but also enriched the research system of college students’ continuance learning intention.

Second, the related factors of flow experience had been introduced into the S-O-R model for testing. Flow experience belongs to the field of psychology. In this study, two elements in the process of flow experience had been introduced, namely, cognitive interest and self-efficacy. The study verified the positive impact of these two elements on college students’ continuance learning intention in DA technology courses, and further affirmed the promoting effect of cognitive interest on self-efficacy. Put simply, this study expanded the research boundary of flow theory and enriched the theoretical basis of antecedent research on continuance learning intention.

Finally, the study introduced self-efficacy into the research model and verified the moderating role. Specifically, it clarified the moderating role of self-efficacy, and deepened the understanding of the relationship between interactivity and continuance learning intention. It enriched the extant literature on the regulating role of self-efficacy, providing a theoretical basis for the construction and reform of DA technology courses in colleges while laying a realistic foundation for the larger community to broadly train talents with DA ability.

### 6.3. Practical implications

The key enlightenment of this study on the curriculum construction and reform of DA technology courses in colleges and universities mainly includes three aspects:

First, cognitive interest is the main factor that affects college students’ continuance learning intention. When colleges and universities offer DA technology courses, attention should be paid to the flow experience of college students, especially their interest in learning. It is highly recommended to implement the teaching method based on up-to-date social cases so that by solving real-life social problems, learners will have a better understanding of the content and the importance of the course; it is also recommended to provide new media resources that are popular among college students to help them understand intuitively the application of big data so that they will no longer have the feeling that data are far from reality and difficult to manage or encapsulate. In other words, using concrete and/or real-world examples to applying big data concepts will ultimately transform learning, making it more interesting and easier to understand. In addition, through increasing industry-university cooperation and collaborative education, the professional elites of enterprises in related fields can be incorporated into the classroom to teach part of the practical content of the course, improving students’ ability to solve practical problems, and further enhancing their cognitive interest.

Second, the external stimuli that college students perceive affect their continuance learning intention through internal factors. The theory that external causes work through internal causes has been repeatedly proved in academia. The findings of this study showed that it is necessary to clearly define the effect path and mechanism of internal v. external factors on students’ continuance learning intention. Teachers of DA technology courses should put student-centered learning to meaningful use, thereby enhancing the awareness of active learning of college students, and improving their ability to be realistic and innovative. In addition to paying attention to internal factors such as the cognitive interest and self-efficacy of college students, instructors should also understand the antecedents of these two elements. This research clarified that measures need to be proposed from three aspects: content quality, software quality, and interactivity.

Third, colleges and universities must emphasize establishing college students’ self-efficacy when offering DA technology courses. Self-efficacy can significantly affect college students’ continuance learning intention and may have a significant moderating role on the relationship between interactivity and continuance learning intention. The role of self-efficacy is therefore prominent and diverse. Colleges and universities can enhance college students’ sense of self-efficacy by optimizing curriculum design, improving software applicability, and enhancing learning interactivity. In the case of insufficient self-efficacy, strengthening interactivity is a supplementary solution. Overall, the goal of improving college students’ continuance learning intention can ultimately be achieved *via* multiple measures.

### 6.4. Limitations

Despite the aforementioned theoretical contributions and practical implications, this study had limitations. First, the sample size was not sufficient, although it meet the basic requirements. Future studies will expand the scope of the investigation to further enhance the reliability of the data analysis. Second, instead of longitudinal data, the cross-section data was used. Therefore, the development process of the research objects cannot be observed. Future research should further be attempted *via* the use of longitudinal research and deepen the discussion of college students’ continuance learning intention from different perspectives.

## 7. Conclusion

In today’s digital era, the demand for DA talents is accelerating daily. However, college students’ continuance learning intention in DA technology courses is challengingly complex and can be easily weaken without a proper and empowering curricular design. Hence, research is needed to further explore the relationship between (S) external stimuli, (O) internal factors and (R) continuance learning intention.

We can draw the following key conclusions. First, among the (S), content quality, software quality, and interactivity had significant positive effects on self-efficacy, whereas the effect of software quality on cognitive interest might not be significant but further investigation is warranted. Second, among (O), cognitive interest and self-efficacy had significant positive effects on continuance learning intention, and cognitive interest had a significant positive effect on self-efficacy. Third, self-efficacy had a significant moderating role on the relationship between interactivity and continuance learning intention. This study had provided new evidence that content quality, software quality, and interactivity had significant positive effects on self-efficacy; in turn, self-efficacy had a significant moderating role on the relationship between interactivity and continuance learning intention. To conclude, this effort is just the beginning of many more future explorations on how cognitive interests and the role of self-efficacy may and can impact college and university students’ learning of DA technology courses to meet the needs for related talents in big data analytics, self-automated devices and related artificial intelligence (AI) domains.

## Data availability statement

The raw data supporting the conclusions of this article will be made available by the authors, without undue reservation.

## Ethics statement

The studies involving humans were approved by Ethics Committee for Scientific Research of Wuhan Business University. The studies were conducted in accordance with the local legislation and institutional requirements. The participants provided their written informed consent to participate in this study.

## Author contributions

LL, PY, and JT contributed to the study conception and design. LL and PY performed the material preparation, data collection, and analysis. All authors contributed to the article and approved the submitted version.

## Funding

This research was supported by Industry-University Cooperative Education Project of Higher Education Department of Ministry of Education (Project No. 220501363301717). Industry-University Cooperative Education Project of Higher Education Department of Ministry of Education (Project No. 202002080012). Philosophy and Social Sciences Research Project of Hubei Provincial Department of Education (Project No. 21G107).
